# Reliability and Validity of the Affect Regulation‐Based Resilience Scale (ARRS): Complementing Coping and Emotion‐Regulation Approaches

**DOI:** 10.1002/pchj.70018

**Published:** 2025-06-03

**Authors:** Xuebing Wu, Jiabao Su, Linlin Yan, Jianhui Wu, Yiqun Gan

**Affiliations:** ^1^ School of Psychological and Cognitive Sciences, Beijing Key Laboratory of Behavior and Mental Health Peking University Beijing China; ^2^ Center for Brain Disorders and Cognitive Science Research Shenzhen University Shenzhen China

**Keywords:** coping, emotion regulation, mental health, resilience, scale development, stress

## Abstract

The Affect Regulation‐based Resilience Scale (ARRS) was developed as an integrative tool to assess adults' psychological resilience. Utilizing a two‐phase approach, the process consisted of item generation followed by rigorous psychometric evaluation. Initial interviews informed item selection, which subsequent analyses including confirmatory factor analysis and validity and reliability analysis using two adult samples (*n* = 424 and *n* = 425). Criterion‐related validity was established by examination relationships between the ARRS, and key constructs: psychological resilience, stress‐related growth, emotion regulation, coping, depression, anxiety, stress, and subjective well‐being. The scale was developed through theoretical and empirical validation, identifying four dimensions (inner resources and goal orientation, positive stress mindset, self and life evaluation, and sensitivity) and comprising 34 validated items. Results indicated satisfactory item performance and good fit for the four‐factor model. The ARRS demonstrated significant positive correlations with psychological resilience, stress‐related growth, cognitive reappraisal, and subjective well‐being, while showing negative correlation with depression, anxiety, expressive suppression and stress*r*. By integrating coping and emotion‐regulation approaches, the ARRS represents a psychometrically robust measure for assessing adults' psychological resilience.

## 1 Introduction

Exposure to adversity and stress constitutes a significant focus in global research, with stress operationally defined as an external or internal stimulus that disrupts homeostasis equilibrium (Chrousos 2009). Stress is associated with a host of adverse outcomes, such as an increased risk of the common cold, cardiovascular disease, obesity, symptoms of depression, and anxiety. However, theorists and researchers have increasingly scrutinized the positive effects of stressful life events. People who experience stressful life events report positive changes in self‐concept, relationships with social networks, personal growth, and life priorities, and having personal resources is key to making these positive changes happen. These adaptive outcomes are not directly caused by stress itself but emerge through the activation of resilience (Lü et al. 2016). In other words, while stress acts as a trigger, individual differences in resilience determine whether its effects manifest as maladaptation or constructive development. For instance, resilience shields against adverse childhood experiences, reducing the risk of mental health issues and fostering positive development in youth (Herrman et al. 2011; Masten et al. 2021).

### 1.1 Exploring Theories, Research Methods, and Constructs in Resilience

The most cited theory in the resilience literature is the meta‐theory of resilience and resiliency, which considers a wide range of ideas and can be applied to different types of adversities at various levels of analysis (e.g., individuals, families, and communities; Fletcher and Sarkar 2013). This theory summarizes three waves of resiliency inquiry, with the first wave focusing on the resilient qualities of individuals and support systems that can predict social and personal success; the second wave concerning the process of coping with stressors or opportunities in a manner that results in the enrichment of protective factors; and the third wave concentrating on the multidisciplinary identification of motivational forces within individuals and how to foster the activation and utilization of these forces (Richardson 2002). Liu et al. (2017) further developed a multisystem model of resilience that recognizes it as an intra‐individual, interpersonal, and socio‐ecological variable.

Studies on resilience reflect little consensus about its conceptualization. Bonanno et al. (2015) argued that resilience refers to temporal elements, such as baseline or pre‐adversity functioning, actual aversive circumstances, post‐adversity resilient outcomes, and the predictors of resilient outcomes. Another review summarized five macro‐categories of resilience: recovery ability, individual functioning, bouncing back capacity, dynamic evolution over time, and positive life adaptation (Sisto et al. 2019). Den Hartigh and Hill (2022) conceptualized resilience as the ability to withstand stressors, rebound, and thrive from them. Troy et al. (2023) recently proposed a more comprehensive conceptualization of resilience that considers adversity, positive adaptation, level of analysis, nature, and criteria for functioning, trajectory, and duration.

### 1.2 Integrative Affect‐Regulation Framework for Resilience

There are two major research perspectives on resilience: the stress and coping perspective and the emotion and emotion‐regulation perspective (Troy et al. 2023). Fletcher and Sarkar (2013) found that most studies conceptualized resilience to adversity and positive adaptation. The *stress and coping perspective* focuses on interactions between individuals and their environment regarding real stressors, responses across various dimensions, coping strategies, and environmental control. It emphasizes the influence of perceived situational demands and personal resources, encompassing internal factors like cognition, optimism, and sense of control, as well as external support such as social networks and life skills (Matheny et al. 1986; Shoda et al. 1993). The *emotion regulation perspective* focuses on emotional response, underlying mechanisms, psychological well‐being, and short‐term reactions in resilience. It places less emphasis on the environment and is closely tied to functional adaptation (Troy and Mauss 2011).

In resilience research, the stress‐coping approach has limitations, particularly its tendency to categorize stressors and stress responses as broadly negative in valence (Lazarus 2000), while neglecting distinct emotional states (Lerner et al. 2015). For instance, fear and anger are both classified as negative emotions, yet they lead to different behavioral responses, highlighting the limitations of relying solely on valence to classify emotions. Additionally, measuring coping abilities is challenging due to the broad spectrum of coping strategies. Conversely, the emotion‐regulation approach overlooks contextual interactions and long‐term changes beyond emotions. The lack of integration and dialogue between these two approaches further compounds these limitations (Compas et al. 2017; John and Eng 2014), hindering the consolidation of resilience research.

To address these limitations, both perspectives can be integrated by emphasizing their shared theoretical foundation—particularly their focus on affect. Affect is a broad, overarching concept that encompasses stress responses, emotions, and other phenomena such as impulses and mood (Compas et al. 2017; Epel et al. 2018; Gross 2015; Marroquín et al. 2017). The stress‐coping approach aims to regulate stress responses, while the emotion‐regulation approach focuses on emotions—both of which can be conceptualized as affect regulation. Troy et al. (2023) proposed an integrative affect‐regulation framework that effectively combines these two perspectives. This framework addresses key limitations by categorizing affect regulation strategies into four types: situation change, attentional deployment, cognitive change, and response modulation (Troy et al. 2023). This classification helps manage the broad range of coping strategies within the stress‐coping perspective. Furthermore, the framework extends the focus beyond short‐term consequences to include not only affective experience but also social processes, affective behavior, physiology, cognitive effort, and engagement (i.e., awareness, behavioral engagement, and learning from adversity). It also accounts for the interactions between these consequences (Troy et al. 2023), offering solutions to the neglect of discrete emotional states in stress‐coping research and the long‐term focus of the emotion‐regulation perspective. Additionally, the framework incorporates environmental interactions, addressing both small‐scale adversity—such as adversity intensity, timing, duration, controllability, affected life domains, and threat/deprivation dimensions—and broader contextual factors, including culture, society, community, social groups, and family (Troy et al. 2023). This resolves the gap in emotion‐regulation research regarding contextual interactions. Therefore, integrating the stress‐coping and emotion‐regulation perspectives into an affect‐regulation framework leverages their complementary strengths to enhance our understanding of mental resilience (Troy et al. 2023).

### 1.3 Scale Development Foundation

Building on the integrated affect‐regulation framework proposed by Troy et al. (2023) and insights from psychological counseling experience, this study conceptualizes resilience from six perspectives:

1. Stress Appraisal and Reappraisal: This category aligns with the framework's focus on affect regulation strategies. Cognitive reappraisal, a key regulation strategy (Gross 2015), plays a crucial role in assessing and reinterpreting stressful situations. The ability to appraise and reappraise stressors determines appropriate emotional and behavioral responses, which are essential for managing stress and fostering resilience (Folkman and Moskowitz 2000).
2. Emotions and Their Regulation: Emotion regulation is central to resilience (Aldao et al. 2010) and a primary focus in counseling. Clients are taught to identify, understand, and modify their emotional reactions to stress (Gross 2015). Effective emotional regulation enables individuals to maintain psychological well‐being despite adversity. This category reflects the active processes of affect regulation strategies.
3. Cognitive and Behavioral Capacity Under Stress (Problem‐Solving Ability): Problem‐solving ability is fundamental in overcoming challenges and preventing emotional distress (Parker and Endler 1992). This category captures the importance of cognitive flexibility and the capacity to take appropriate actions in response to adversity, linking to short‐term consequences outlined in the theoretical framework.
4. Avoidance, Distraction, Detachment, Surrender, and Acceptance: These coping strategies, which involve disengagement or detachment from stressors, can be adaptive or maladaptive depending on the context (Aldao et al. 2010). In counseling, it is essential to help clients recognize when avoidance may increase distress and when acceptance is a necessary strategy for long‐term resilience (Hayes et al. 2006). This category emphasizes temporary disengagement and eventual acceptance, aligning with the short‐term consequences aspect of the integrated framework.
5. Psychological Resources under Stress—Social Support (External Resources): Social support is a crucial factor in resilience (Cohen and Wills 1985). In counseling, strong social networks are emphasized, as individuals who feel supported by others tend to exhibit greater resilience (Thoits 2011). This category highlights the contextual interaction component of the integrated framework.
6. Psychological Resources Under Stress—Internal Resources (Control, Self‐Efficacy, Intrinsic Motivation, Optimism, Meaning in Life): Internal resources such as self‐efficacy, optimism, and a sense of meaning are essential for effective stress coping (Bandura 1997; Ryff 2014). These internal resources significantly contribute to resilience by fostering psychological well‐being, even in challenging circumstances. This category reflects affect regulation strategies, short‐term consequences, and contextual interaction within the integrated framework.

These six perspectives not only highlight the structure of the integrated affect regulation framework but also serve as the foundation for developing the new scale for resilience assessment.

The earliest resilience scale was developed by Wagnild and Young (1993) and is centered solely on two dimensions: personal competence and acceptance of self and life (Ryff 2014). Wagnild further developed a short version—the 14‐item Resilience Scale (RS14)—with five dimensions: meaningfulness of life, perseverance, self‐esteem, composure, and loneliness (Wagnild 2011). Further, the Connor‐Davidson Resilience Scale (CD‐RISC), developed by Connor and Davidson (2003), is the most widely used scale of resilience. It consists of five dimensions: personal competence, trust, positive acceptance, control, and spiritual influence (Connor and Davidson 2003), but lacks consideration of personal resources. Smith and colleagues designed the Brief Resilience Scale (BRS) for process‐oriented resilience with four dimensions: personal traits, social relationships, coping, and health (Smith et al. 2008). However, the BRS lacks a measure of emotion regulation. In 2011, Dai and colleagues designed a resilience scale with four dimensions: problem solving, social support, self‐confidence, and positive cognition (Dai et al. 2011); however, it lacks involvement of emotional regulatory functioning.

In summary, current resilience scales are still not sufficient to obtain a more comprehensive understanding of resilience. Therefore, this study utilizes previous resilience scales and the affect‐regulatory framework proposed by Troy et al. (2023) to design a new resilience scale: the Affect Regulation‐based Resilience Scale (ARRS).

### 1.4 The Current Study

Considering the potential shortcomings of existing psychological resilience scales, there is a critical need for more comprehensive measures. Consequently, the main objective of this study was to develop a new resilience scale that complements coping and emotion‐regulation approaches with the following dimensions: (1) stress appraisal and reappraisal, (2) emotions and their regulation, (3) cognitive and behavioral capacity under stress (problem‐solving ability), (4) avoidance, distraction, detachment, surrender, and acceptance (learning to accept and let go of unmanageable situations), (5) psychological resources under stress—social support (external resources), and (6) psychological resources under stress—internal resources, including control and self‐efficacy, intrinsic motivation, optimism, and a sense of meaning in life. The Affect Regulation‐based Resilience Scale (ARRS) was developed and validated through the following steps: (1) identification of the item pool through interviews, (2) item determination, and (3) measurement testing.

## 2 Method

### 2.1 Participants

Participants were recruited online through convenience sampling. The participant inclusion criteria included no history of mental illness and high school education or above.

Sample 1 was used for the *initial analysis* in developing the new measures, and 424 valid questionnaires were collected after excluding participants who inaccurately completed lie detection and attentional questions, responded in under 600 s, were under 18 years old, or provided repeated answers. Participants were aged 18–58 years (*M*_age_ = 26.98 ± 6.64 years) and 54.25% were women. Among the participants, 58.7% held a bachelor's degree, followed by 16.5% with a master's degree. Those with college diplomas accounted for 15.6%, while 5.0% had doctoral degrees and 4.2% completed high school or less. Students constituted the largest group (32.8%), while office staff and professionals represented 20.0% and 17.9%, respectively. Managers comprised 13.2%, with the remaining 15.8% working in combined fields including healthcare, service industries, and technical roles.

Sample 2 was used for *measurement testing*, and 425 valid questionnaires were collected. Participants were aged 18–54 years (*M*_age_ = 26.6 ± 6.76 years) and 52.2% were women. The cohort comprised 67.3% bachelor's degree holders, 13.2% master's graduates, and 12.2% with associate degrees. Those with doctoral degrees constituted 1.9%, while 5.4% had completed secondary education or lower. Full‐time students represented the majority (35.1%), followed by professionals (17.4%) and administrative staff (14.8%). Corporate executives accounted for 12.2%, with the remaining 20.5% distributed among service industries, healthcare, production roles, and other occupations.

### 2.2 Qualitative Analysis

To develop the ARRS, we first recruited 21 adults aged 20–49 years for semi‐structured online interviews using the snowball method. Participants were selected based on self‐identified or peer‐identified psychological resilience levels, assessed through two items evaluating their ability to handle prolonged stress and critical situations. These items were rated on a 10‐point scale, ranging from 1 (*Very Poor*) to 10 (*Excellent*). High‐resilience individuals were those who maintained a positive mindset during prolonged stress and performed exceptionally well in critical situations, such as exams, public speaking, competitions, or emergencies. In contrast, low‐resilience individuals struggled to maintain a positive mindset under stress and often underperformed in key moments. To ensure validity, only participants with clearly high or low resilience levels were included, while those with moderate resilience were excluded. The final sample consisted of seven medical workers, three medical students, three athletes, two militaries, and six individuals from other professions (*M*_age_ = 28.45 ± 6.85 years; 12 women and 9 men). A semi‐structured interview outline was developed based on existing literature and resilience scales. Twelve psychology graduate students/professors were recruited to conduct one‐on‐one interviews with high‐ and low‐resilience, as well as their recommenders (the first responders). The interview collected information on participants' backgrounds, instances demonstrating high or low resilience, typical stress‐inducing events, and relevant personal qualities (see Supporting Information). The interview process incorporated a coding process developed through expert discussions. It subjectively evaluated high‐resilience individuals under daily stress, focusing on their responses, behaviors, external influences, personal traits, coping strategies, and the impact of stress. All interviews were recorded, and transcripts were verified post‐interview to ensure accuracy.

The qualitative analysis of interview content I followed the method proposed by Hu and Gan (2008). First, a hierarchical categorization process was applied to the qualitative data, with categorical analysis used to code the coping processes described by respondents. The interview transcripts were initially categorized based on six dimensions: (1) stress appraisal and reappraisal, (2) emotions and their regulation, (3) cognitive and behavioral capacity under stress (problem‐solving ability), (4) avoidance, distraction, detachment, surrender, and acceptance (learning to accept and let go of unmanageable situations), (5) psychological resources under stress—social support (external resources), and (6) psychological resources under stress—internal resources, including control and self‐efficacy, intrinsic motivation, optimism, and a sense of meaning in life. For ambiguous or unclear response, independent coding was conducted to interpret their meaning before assigning them to categories. To enhance the accuracy of the categorization process, interviewees provided feedback to confirm that the assigned categories accurately reflected their experiences. Additionally, third‐party experts who were not involved in this study reviewed the process to assess its accuracy and minimize potential bias.

### 2.3 Measures

#### 2.3.1 Demographics Questionnaire

All participants answered demographic questions concerning the following information: age, sex, education level, and occupation.

#### 2.3.2 Lie Detection and Attentional Questions

Ten lie detection and attentional questions were evenly interspersed among all questionnaires: lie detection questions (e.g., “I have never seen rain”) and attentional questions (e.g., “Please select strongly disagree for this question”).

#### 2.3.3 ARRS

ARRS was scored on a five‐point scale (1 = *complete non‐alignment* and 5 = *complete alignment*). Cronbach's alpha was 0.955 in this study, indicating excellent internal consistency.

#### 2.3.4 Measures of Criterion Validity

*The short version of the Psychological Resilience Scale*. This 10‐item scale, designed to assess resilience in response to stress or trauma, has been validated for its structure and reliability among adults affected by earthquakes in China (Wang et al. 2010). It is scored on a five‐point scale (1 = *never* to 5 = *almost always*). In the sample 2, Cronbach's alpha was 0.892, indicating good internal consistency.

*Stress‐related Growth Scale*. The reliability of this scale was validated in the Chinese population (Li et al. 2018). This 15‐item scale assesses personal growth following a stressful event, measuring both interpersonal (eight items) and intrapersonal (seven items) dimensions. It is scored on a five‐point scale (1 = *a very poor fit* to 5 = *a very good fit*). In sample 2, Cronbach's alpha was 0.937, indicating excellent internal consistency.

*Emotional Regulation Questionnaire*. This 10‐item scale, translated into Chinese and validated by Chen et al. (2020), measures emotional regulation strategies. The scale includes two subscales: Cognitive Reappraisal (6 items), a positive regulation strategy where individuals modify their emotions by altering their external environment or internal psychological state, and Expressive Inhibition (4 items), a negative regulation strategy where individuals adjust their emotional responses by changing their behavioral reactions to either diminish or enhance their emotional experience. Responses were scored on a seven‐point scale, ranging from 1 (*complete disagreement*) to 7 (*complete agreement*). In sample 2, Cronbach's alphas were 0.855 for cognitive reappraisal and 0.797 for expressive inhibition, indicating good internal consistency for both subscales.

*The Emergency Coping Ability Scale (ECAS)*. Developed by Yang and Zhao (2012), this 15‐item scale was originally designed for university student populations and later validated in a community resident sample in China. The scale uses a five‐point Likert format, where 1 indicates “*strongly disagree*” and 5 indicates “*strongly agree*”. It assesses adults' ability to cope with emergencies from three perspectives: cognition, emotion, and behavior. The scale includes three dimensions: positive emotion (7 items), negative emotion (4 reverse‐coded items), and positive behavior (4 items). In sample 2, Cronbach's alpha was 0.909, demonstrating excellent internal consistency.

*Subjective well‐being*. This five‐item scale, translated from Bonsignore et al. (2001), measures participants' overall subjective well‐being over the past 2 weeks. The scale has been validated in the Chinese population (Fung et al. 2022). Responses are scored on a six‐point scale (0 = *never* to 5 = *all the time*), with higher scores indicating more happiness. In sample 2, Cronbach's alpha was 0.929, indicating excellent internal consistency.

*Depression–Anxiety–Stress Self‐rating Scale*. The Chinese version of this scale has been previously validated (Wang et al. 2016). This 21‐item scale is divided into three subscales (seven items each) to measure symptoms of depression, anxiety, and stress over 1 week. The items are rated on a four‐point scale (0 = *no match* to 3 = s*trong match*), with higher scores indicating more severe symptoms. In sample 2, Cronbach's alphas were 0.890 for depression, 0.873 for anxiety, and 0.869 for stress, indicating good to excellent internal consistency across the subscales.

### 2.4 Procedures

This study was approved by an appropriate review board of Peking University, and informed consent forms were signed by the interviewees prior to the interviews and surveys. The interview duration ranged from 20 to 60 min. For the initial analysis and the measurement testing, participants in both samples completed the following self‐report questionnaires: demographics questionnaire, lie detection and attentional questions, the short version of the Psychological Resilience Scale, the Stress‐related Growth Scale, the Emotional Regulation Questionnaire, the Subjective Well‐being Scale, and the Depression–Anxiety–Stress Self‐rating Scale. The entire survey lasted approximately 20 min and was administered through the Questionnaire Star (https://www.wjx.cn/) online survey platform. Participants received ¥5 for their participation.

### 2.5 Data Analysis

There were no missing values or outliers in Sample 1. Data validity was initially assessed, followed by item analysis, including examination of item discrimination, item‐total correlations, and an exploratory factor analysis. Data analysis was performed using SPSS version 24.0 and R. Items with item‐total correlations less than 0.4 were deleted. The raw data and the confirmatory factor analysis data can be accessed via the link https://osf.io/3jnvp/?view_only=f32928912aee4a9a9bf20e2077c4747e.

No missing values or outliers were found in Sample 2. Initially, we evaluated the internal consistency and validity of the ARRS. Then, structural validity was assessed via confirmatory factor analysis using R software. We conducted the confirmatory factor analysis (CFA) using the Diagonally Weighted Least Squares (DWLS) method (Li 2016). Model fit was evaluated based on widely accepted criteria. Specifically, values of the Comparative Fit Index (CFI) and Tucker‐Lewis Index (TLI) greater than or equal to 0.90 indicate good fit, while values greater than or equal to 0.95 reflect excellent fit. A Root Mean Square Error of Approximation (RMSEA) value below 0.05 indicates excellent model fit, and a Standardized Root Mean Square Residual (SRMR) value below 0.08 is considered indicative of good fit (Hu and Bentler 1999). The reliability and external validity of the scales were analyzed using SPSS 24.0. Cronbach's *α* coefficients were separately computed for the total score and scores for each dimension of the ARRS. Correlation coefficients were employed to measure the correlations between the aforementioned scores and those obtained from the short version of the Psychological Resilience Scale, the Stress‐related Growth Scale, the Emotional Regulation Questionnaire, the Subjective Well‐being Scale, and the Depression–Anxiety–Stress Self‐Rating Scale. This was performed to verify the external validity of the scale. Significance was set at 0.05.

## 3 Results

### 3.1 Qualitative Results

The original materials were categorized into the following dimensions: (1) stress appraisal and reappraisal, (2) emotions and their regulation, (3) cognitive and behavioral capacity under stress (problem‐solving ability), (4) avoidance, distraction, detachment, surrender, and acceptance (learning to accept and let go of unmanageable situations), (5) psychological resources under stress—social support (external resources), and (6) psychological resources under stress—internal resources, including control and self‐efficacy, intrinsic motivation, optimism, and a sense of meaning in life. We then outlined differences among individuals with different resilience levels in these categories under stress. Subsequently, a trained coder organized participants' typical expressions into key elements and named these key elements. Based on the literature review and interview content, the researchers developed an initial version of the item pool. After discussions with nearly 10 experts in the field, the ARRS comprised 126 items, including 63 reverse‐scored items.

For Sample 1, after analyzing item discrimination and item‐total correlation and deleting items with item‐total correlation below 0.4, the total correlation of the remaining items was between 0.400 and 0.764. Exploratory factor analysis was subsequently conducted. Bartlett's spherical test and the Kaiser–Meyer–Olkin (KMO) test were performed first, and the Bartlett's spherical test statistic was 33,353.98 (KMO = 0.970 > 0.90, and *p* < 0.001, respectively), which is suitable for factor analysis. Subsequently, a principal axis factor analysis was conducted. The eigenvalues of the first 15 factors were greater than 1; however, the eigenvalues of the first four factors were greater than 2 and collectively explained 50.71% of the variance, and the inflection point in the scree plot was the fourth factor. According to the principle of “removing items with communalities less than 0.5, null loadings, and double loadings,” 67 items were deleted, resulting in 17 items in Factor I, 9 items in Factor II, 10 items in Factor III, and 6 items in Factor IV (*n* = 42 items). Subsequently, owing to item similarity, 7 items from Factor I and 1 item from Factor III were removed, resulting in 10 items for Factor I, 9 items for Factor II, 9 items for Factor III, and 6 items for Factor IV (*n* = 34 items). The four factors explained 63.09% of the variance: Factor I = 46.57%, Factor II = 8.80%, Factor III = 4.63%, and Factor IV = 3.09%.

Factor I, Inner Resources and Goal Orientation (Cronbach's *α* = 0.915), measures an individual's internal belief foundation, intrinsic motivation‐driven goal pursuit, and the ability to maintain goal orientation under pressure. Factor II, Positive Stress Mindset (Cronbach's *α* = 0.888), focuses on individuals' psychological response to stress, including embracing challenges, viewing stress as a potential stimulant, maintaining confidence in adversity, handling issues calmly, and adopting a long‐term perspective to transform stress into growth opportunities. Factor III, Self and Life Evaluation (Cronbach's *α* = 0.920), focuses on an individual's subjective assessment of self and life state, evaluating self‐criticism, feelings of being wronged, and overall psychological well‐being, including self‐perception, life satisfaction, and the evaluation of personal achievements and quality of life. Factor IV, Sensitivity (Cronbach's *α* = 0.933), captures individuals' sensitivity to stress, encompassing physical and emotional tension, self‐blame behaviors, and negative experiences. This factor reflects the vulnerability of psychological defenses and the extent of emotional fluctuations under stress. The Cronbach's *α* of the total scale was 0.964, indicating excellent internal consistency, and the item‐total correlations ranged from 0.517 to 0.762. The ARRS comprised 34 item (see the Supporting Information). The factor loading, discriminant validity, and total correlations for each item are presented in Table 1.

**TABLE 1:** The factor loadings, discrimination, and total question correlation for the ARRS scale.

Factor	Item	Loading	Discrimination	Total question correlation
Inner Resources and Goal Orientation	Q4	0.77	0.38	0.63
	Q5	0.72	0.37	0.68
	Q2	0.72	0.26	0.52
	Q6	0.66	0.32	0.60
	Q7	0.65	0.26	0.55
	Q9	0.64	0.28	0.59
	Q3	0.60	0.35	0.62
	Q8	0.50	0.36	0.70
	Q1	0.46	0.32	0.62
Positive Stress Mindset	Q15	−0.70	0.37	0.63
	Q12	−0.63	0.34	0.66
	Q10	−0.61	0.34	0.55
	Q13	−0.59	0.34	0.70
	Q11	−0.55	0.30	0.58
	Q14	−0.54	0.37	0.65
Self and Life Evaluation	Q18	0.74	0.46	0.66
	Q24	0.69	0.44	0.64
	Q19	0.63	0.52	0.67
	Q23	0.61	0.51	0.68
	Q16	0.54	0.44	0.65
	Q17	0.52	0.51	0.70
	Q20	0.48	0.37	0.70
	Q21	0.47	0.51	0.69
	Q22	0.36	0.52	0.66
Sensitivity	Q29	0.81	0.60	0.71
	Q32	0.81	0.57	0.76
	Q30	0.75	0.54	0.63
	Q31	0.72	0.55	0.69
	Q28	0.70	0.62	0.70
	Q34	0.66	0.55	0.67
	Q25	0.63	0.58	0.71
	Q26	0.57	0.60	0.70
	Q27	0.54	0.56	0.70
	Q33	0.47	0.55	0.72

### 3.2 Descriptive Statistics

Table 2 displays the means and standard deviations of total and dimensional resilience scores among individuals of different genders in Sample 2. Independent sample *t*‐tests revealed gender differences only for the dimensions of Inner Resources and Goal Orientation (*t* = 2.465, *p* = 0.014) and Positive Stress Mindset (*t* = 3.006, *p* = 0.03), with men scoring higher than women. No significant differences were observed in total or dimensional scores based on individuals' educational backgrounds.

**TABLE 2:** Means and standard deviations of total ARRS scores and scores of each dimension for individuals of different genders.

	male (*n* = 203)		female (*n* = 222)	
	M	SD	M	SD
Inner Resources and Goal Orientation	4.13	0.55	4.00	0.59
Positive Stress Mindset	4.02	0.60	3.83	0.63
Self and Life Evaluation	3.83	0.92	3.95	0.78
Sensitivity	3.32	0.97	3.26	0.94
Total	128.89	22.58	127.19	21.96

*Note:* The dimensional mean score is the mean value obtained by dividing the total score of each dimension by the number of questions in that dimension.

### 3.3 Measurement Testing

#### 3.3.1 Internal Consistency of the ARRS

Using Sample 2, Table 3 presents the internal consistency of the total ARRS and dimension scores. The ARRS demonstrated excellent overall internal consistency (Cronbach's *α* = 0.955), and the Cronbach's *α*s of the four factors were 0.925, 0.889, 0.909, and 0.843, respectively, also demonstrating excellent internal consistency. Furthermore, the Omega coefficients for the total ARRS and its four subscales were also calculated. The Omega total for the entire scale was 0.97, indicating excellent overall internal consistency. The Omega hierarchical coefficients for the four factors were as follows: Factor I (0.87), Factor II (0.86), Factor III (0.89), and Factor IV (0.92), demonstrating strong internal consistency and a well‐structured hierarchical structure. These findings align with the Cronbach's *α* values, further confirming the reliability of the ARRS and its subscales.

**TABLE 3:** The internal consistency and Spearman correlation coefficients with criterion scales.

	*Cronb*—*ach's α*	The Emergency Coping Ability Scale (ECAS)	Psychological resilience (Short)	Stress related growth	Cognitive reappraisal	Expressive suppression	Depression	Anxiety	Stress	Subjective well‐being
Inner Resources and Goal Orientation	0.889	703**	0.780**	0.715**	0.614**	0.188**	−0.508**	−0.403**	−0.437**	0.722**
Positive stress mindset	0.843	0.686**	0.749**	0.626**	0.600**	0.197**	−0.432**	−0.343**	−0.366**	0.660**
Self and Life Evaluation	0.909	0.579**	0.528**	0.477**	0.365**	−0.186**	−0.666**	−0.624**	−0.647**	0.479**
Sensitivity	0.925	0.655**	0.556**	0.424**	0.316**	−0.125**	−0.650**	−0.668**	−0.677**	0.545**
Total	0.955	0.742**	0.703**	0.589**	0.479**	−0.065	−0.695**	−0.650**	−0.666**	0.644**

*Note:* The Emotional Regulation Questionnaire contains cognitive reappraisal and Expressive suppression.

** *p* < 0.01.

#### 3.3.2 Factor Structure of the ARRS

**FIGURE 1:**
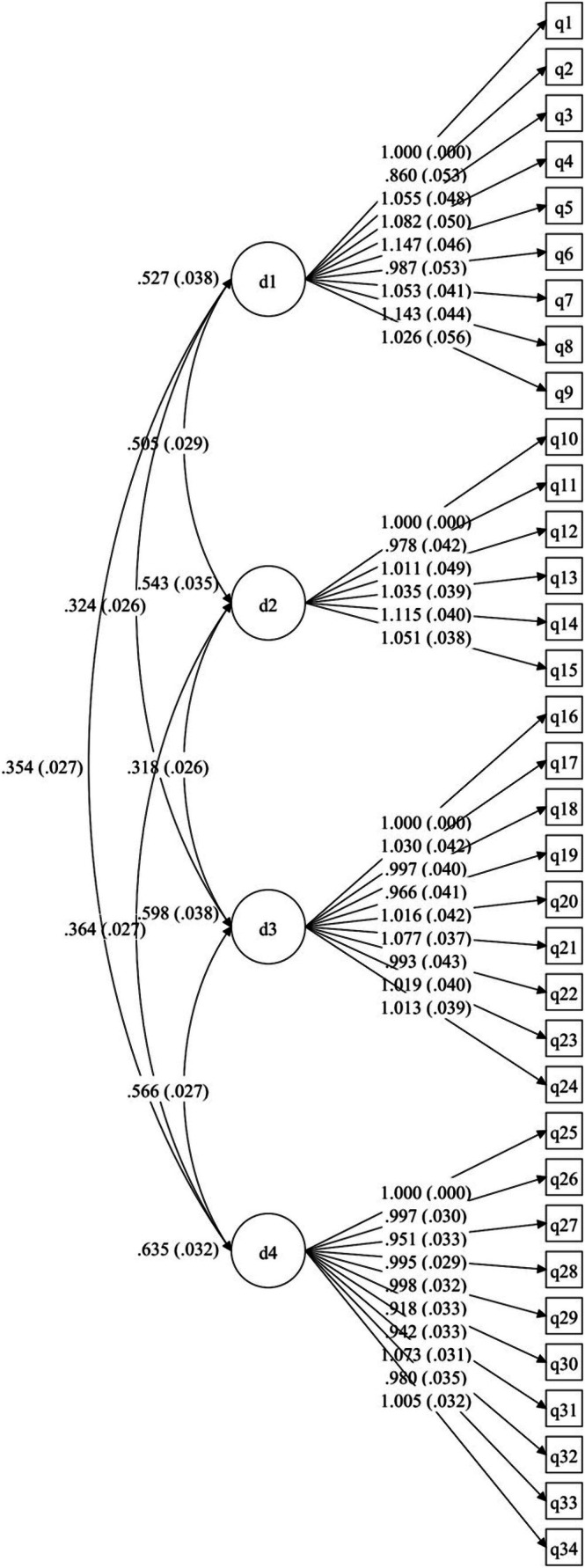
The four‐factor model of resilience. “d1” means Inner Resources and Goal Orientation, “d2” means Positive Stress Mindset, “d3” means Self and Life evaluation, and “d4” means Sensitivity.

Using Sample 2, we conducted a confirmatory factor analysis (Figure 1). All indicators met the criteria (RMSEA = 0.035, SRMR = 0.044, CFI = 0.998, and TLI = 0.997).

#### 3.3.3 Convergent and Discriminant Validity

The four‐factor model demonstrated a good fit for Sample 2 data. Table 4 presents the CR and AVE. The CR for each dimension was greater than 0.7. Although the AVE of Inner Resources and Goal Orientation and Positive Stress Mindset were slightly less than 0.5, the AVE of the remaining dimensions were higher than 0.5. Overall, the four‐factor model of resilience had fair convergent and discriminant validity.

**TABLE 4:** Correlation, composite reliability (CR), and averaged variance extracted (AVE) of the ARRS.

	1	2	3	4
1. Inner resources and goal orientation	—	0.635^a^	0.267^a^	0.522**
2. Positive stress mindset	0.797**	—	0.494**	0.529**
3. Self and life evaluation	0.517**	0.244^a^	—	0.837**
4. Sensitivity	0.272^a^	0.280^a^	0.701^a^	—
Total	0.733**	0.719**	0.901**	0.927**
AVE	0.477	0.476	0.529	0.555
CR	0.890	0.845	0.910	0.926

^a^ The value is the squared value of the correlation coefficient of the two factors.

** *p* < 0.01.

#### 3.3.4 Criterion Validity of the ARRS

Table 3 also presents the Pearson correlation coefficients of the total ARRS and dimension scores with the criterion scale scores for Sample 2. The total and dimensional scores of the ARRS were significantly and positively correlated with the Emergency Coping Ability Scale (ECAS), the Short Version of the Psychological Resilience Scale, Stress‐related Growth Scale, Emotional Regulation Questionnaire (cognitive reappraisal), and the Subjective Well‐being Scale. Both the total and dimensional ARRS scores were significantly and negatively correlated with the Emotional Regulation Questionnaire (expressive suppression), depression, anxiety, and stress. These results supported the criterion validity of the ARRS.

## 4 Discussion

Based on the affect‐regulatory framework proposed by Troy et al. (2023), this study developed a comprehensive scale to integrate the core factors of resilience in response to the limitations of previous resilience scales. Using a sample of adult participants, the final version of the ARRS consisted of 34 items distributed across four factors: Inner Resources and Goal Orientation, Positive Stress Mindset, Self and Life Evaluation, and Sensitivity.

Dimension 1, Inner Resources and Goal Orientation: This dimension captures an individual's foundational psychological resources, including intrinsic motivation and goal‐directed behavior. It aligns with the affect‐regulation framework by encompassing attentional deployment, response modulation strategies, and the cognitive and behavioral consequences of engagement (Troy et al. 2023). Individuals with strong inner resources demonstrate enhanced problem‐solving abilities and persistence under stress, reflecting their capacity to maintain focus and determination (Parker and Endler 1992). Furthermore, this dimension corresponds to the original category of cognitive and behavioral capacity under stress, incorporating avoidance and acceptance coping strategies, as well as internal resources. Personal values and life goals play a crucial role in guiding adaptive responses. It emphasizes self‐efficacy and perceived control, enabling individuals to navigate challenges effectively (Bandura 1997). Unlike the other dimensions, this one focuses on internal strengths and goal orientation, fostering resilience through sustained engagement with life goals. For individuals with low scores in this dimension, targeted interventions could help enhance goal‐setting abilities. For instance, the Goal‐setting and Planning intervention (Coote and MacLeod 2012) may support the development of persistence and the ability to maintain focus on life goals despite stress.

Dimension 2, Positive Stress Mindset: This dimension examines an individual's adaptive psychological response to stress, particularly their ability to reframe stress as a challenge rather than a threat. It integrates stress appraisal and reappraisal processes, emphasizing cognitive reframing, learning from adversity, and the perceived controllability of stress within the affect‐regulation framework (Lazarus and Folkman 1987; Troy et al. 2023). Individuals with a positive stress mindset exhibit confidence and composure during adversity, viewing stress as an opportunity for growth. This dimension aligns with the original category of stress appraisal and reappraisal, highlighting the role of adaptive cognitive interpretations in fostering resilience (Crum et al. 2013). Unlike Inner Resources and Goal Orientation, which emphasizes strategic action, this dimension focuses on perception and mindset in relation to stress. For individuals with low scores in this dimension, targeted interventions may help reshape their stress perceptions. A metacognitive approach (Crum et al. 2023) can be particularly effective in guiding individuals to reframe stress as an opportunity for growth, thereby reducing the tendency to perceive stress as a threat.

Dimension 3, Self and Life Evaluation: This dimension captures subjective appraisals of oneself and life circumstances, reflecting overall self‐perception and life satisfaction. It aligns with the affect‐regulation framework by incorporating short‐term emotional consequences and cognitive efforts related to self‐reflection (Troy et al. 2023). Individuals who engage in positive self‐evaluation tend to experience greater well‐being and resilience. Rooted in theories of self‐concept and self‐acceptance (Ryff 2014), this dimension underscores the role of positive self‐assessment in maintaining psychological health. Additionally, it indirectly reflects the original category of external resources, as social support plays a crucial role in fostering a positive self‐view (Rees and Freeman 2007). Unlike Positive Stress Mindset, which focuses on stress perception, this dimension evaluates broader aspects of self‐worth and life satisfaction beyond immediate stress contexts. For individuals with low scores in this dimension, targeted interventions can help enhance self‐worth and life meaning. Meaning‐making interventions (Park 2010) may be particularly effective in strengthening self‐esteem and promoting overall life satisfaction.

Dimension 4, Sensitivity: This dimension assesses emotional and physiological responses to stress, capturing heightened reactivity, self‐blame, and the prolonged impact of stressful experiences. It reflects the physiology and emotional components of the affect‐regulation framework (Troy et al. 2023). High sensitivity indicates a tendency toward intense emotional reactions, aligning with the original category of emotions and their regulation. Theories of allostatic load (McEwen 1998) support this dimension by explaining how chronic stress exposure contributes to cumulative physiological burden. Unlike the other dimensions, which focus on adaptive strategies and mindset, Sensitivity highlights an individual's interaction with adversity, emphasizing vulnerabilities in resilience. For individuals with low scores in this dimension, interventions can focus on mindfulness‐based approaches (Creswell et al. 2019) to enhance emotional awareness and reduce the physiological impact of stress. Beyond its theoretical significance, this scale has practical applications in identifying distinct response patterns and coping strategies under stress. By covering emotional, cognitive, and behavioral aspects, it provides a comprehensive understanding of individual coping mechanisms, offering valuable insights for designing targeted interventions to enhance resilience.

The Wagnild and Young resilience scale (RS) and its shortened version, RS‐14, primarily assess individual traits (Wagnild 2011; Wagnild and Young 1993). However, they exhibit ambiguity regarding whether resilience is a unidimensional or multidimensional construct (Ahern et al. 2006). Furthermore, the administration procedures and scoring details are not well‐documented, and the absence of reverse‐scored items increases the risk of response bias (Ahern et al. 2006). Similarly, the Brief Resilience Scale (BRS) focuses specifically on an individual's ability to bounce back from stress but does not provide a comprehensive assessment of the resilience process (Smith et al. 2008). Additionally, its data reduction process was based on pilot feedback rather than rigorous empirical validation (Windle et al. 2011). The Adult Resilience Scale (Dai et al. 2011), developed within a Chinese cultural context, incorporates dimensions such as problem‐solving, social support, self‐confidence, and positive cognition. However, these dimensions primarily reflect a stress‐coping perspective, with limited consideration of emotion regulation capacities. Most existing resilience measures adopt either a stress‐coping or emotion‐regulation perspective, failing to integrate both aspects comprehensively. Although the Connor‐Davidson Resilience Scale (CD‐RISC) offers broad coverage of personal traits, stress coping, emotion regulation, and interaction with the environment, it remains an individual‐level measure that lacks a clear consideration of social or contextual influences (Connor and Davidson 2003). Additionally, it does not fully capture personal resources such as self‐efficacy and meaning in life, which are crucial for resilience and personal growth (Updegraff and Taylor 2000). Research suggests that greater self‐efficacy and a strong sense of meaning in life are associated with higher subjective well‐being (Krok et al. 2021). The affect‐regulation framework proposed by Troy et al. (2023) provides the most recent and comprehensive perspective on psychological resilience. It emphasizes the integration of stress‐coping and emotion‐regulation by categorizing affect regulation strategies, broadening the scope of short‐term consequences, and incorporating environmental interactions to offer a more holistic understanding of resilience. Currently, no existing resilience scales are specifically designed to comprehensively assess resilience within this framework. Therefore, this study not only provides a more comprehensive assessment compared to prior scales but also contributes to the validation of the affect‐regulation framework.

This study examined the structural validity of the ARRS by exploring the correlations between these factors. Although the AVE of “inner resources” and “positive stress mindset” were slightly less than 0.5, the AVE of the remaining factors was higher than 0.5 and the CR of all factors was higher than 0.7. Overall, the scale had satisfactory structural validity. Furthermore, age and subjective socioeconomic status were positively correlated with the total and all sub‐factor scores. Among them, there were sex differences in the inner resources and goal orientation, and positive stress mindset factors, with men scoring higher than women. This aligns with previous research, in which men tend to have slightly higher self‐esteem scores than women. Perhaps men are more confident about their future in the face of adversity, whereas women tend to be relatively self‐deprecating (Magee and Upenieks 2019).

This study employed Spearman's correlation analysis to examine the criterion validity of the ARRS. Scale scores showed moderate correlations with the criterion that is most desirable (Hu and Gan 2008). The CD‐RISC, a widely used scale, showed correlations of roughly 0.36 and 0.83 with other criterion scales (Connor and Davidson 2003). Another review summarized the correlations among several resilience scales ranging from 0.51 to 0.71 (Windle et al. 2011). The correlations between the total ARRS score and Emergency Coping Ability Scale (ECAS), stress‐related growth, cognitive reappraisal, and life satisfaction were 0.742, 0.589, 0.479, and 0.644, respectively; thus, the validity of the ARRS was satisfactory.

This study complements the strengths and unique contributions of the coping and emotion‐regulation approaches through the affect‐regulation framework (Troy et al. 2023). The ARRS has unique dimensions, such as a positive stress mindset and internal resources, and goal orientation, which provide valuable insights into the dimensions of resilience. A positive stress mindset has been shown to facilitate resilience, particularly among individuals who have experienced childhood maltreatment, by enabling them to reframe adversity as a challenge rather than a threat (Boullion et al. 2021). Similarly, goal orientation plays a crucial factor in resilience, as individuals with a strong focus on goals demonstrate greater persistence and adaptability in the face of challenges (Jowkar et al. 2014). These findings underscore the importance of mindset and inner resources in psychological resilience, offering valuable implications for the development of personalized interventions.

However, several limitations warrant consideration in future research to enhance the applicability and robustness of the scale. First, although the final structure differs from the initial six‐dimensional framework, the four‐factor model largely captures the theoretical and clinical content of the original dimensions. However, external supportive resources (e.g., social support) were not sufficiently represented. This limitation may stem from the challenge of accurately measuring social dimensions through self‐report methods. Future studies could address this gap by incorporating multi‐informant reports or behavioral assessments to provide a more comprehensive evaluation of social resources. Second, retest reliability remains to be assessed, which is critical for establishing the temporal stability of the ARRS. Future research should examine test–retest reliability across various intervals to determine the stability of resilience as measured by the ARRS. Finally, the applicability of the scale across different contexts and age groups requires further exploration. Given that resilience is influenced by developmental stages and cultural backgrounds, it is essential to examine whether the ARRS maintains its factor structure and predictive validity across diverse populations. Future research should conduct cross‐cultural and age‐specific validation studies to refine the scale and ensure its generalizability.

## Ethics Statement

Informed consent was obtained from all participants included in the study. All procedures in studies involving human participants were performed in accordance with the ethical standards of the institutional review board of Peking University. The study was performed in accordance with the ethical standards as laid down in the 1964 Declaration of Helsinki and its later amendments or comparable ethical standards.

## Conflicts of Interest

The authors declare no conflicts of interest.

## Supporting information


Data S1.
