# Supplementary material for: Reliability and Validity of the Affect Regulation‐Based Resilience Scale (ARRS): Complementing Coping and Emotion‐Regulation Approaches
Source: Psych J. 2025 Jun 3;14(4):591–602. doi: 10.1002/pchj.70018 (PMC12318594; doi:10.1002/pchj.70018)
Supplement: Supplementary file 1 — Data S1. [file PCHJ-14-591-s001.docx]

The scale development process—encompassing item generation, psychometric validation, and finalization—was rigorously conducted in Mandarin Chinese to preserve cultural and linguistic authenticity. To enhance global utility, a English translation, is provided in Supplementary Materials. Researchers employing this instrument in English contexts are advised to validate its psychometric properties within their target populations. The original Chinese materials can be accessed via the link <https://osf.io/3jnvp/?view_only=f32928912aee4a9a9bf20e2077c4747e> .

**Semi - structured Interview guideline**

**Part 1: Interviews with Recommenders (Recommenders themselves can skip this part)**

Interview Time:

Interview Location:

Interview Method (Online/Offline):

1. Do you have any acquaintances with typical high or low psychological resilience around you? Who are they? A person with high psychological resilience can maintain a good mindset after encountering difficulties or still perform outstandingly in critical situations such as exams, public speaking, competitions, or emergencies.
2. What is your relationship with them? How well do you know each other (from 1 - not knowing to 9 - being together every day)?
3. Please introduce the basic information of the person: gender, age, educational background, and occupation.
4. Do you think their high/low resilience is manifested in prolonged stress or in critical situations? Can you give an example to illustrate?
5. What do you think is the reason why he/she is so typical? Can you give an example? How is his/her stress - resistance ability? Will he/she be broken down by prolonged hardships? Will he/she fail at critical moments? How is his/her ability to handle critical situations?
6. What kind of qualities do you think he/she has that can help him/her cope with prolonged stress? / What kind of qualities do you think he/she lacks that prevent him/her from coping with prolonged stress?
7. What kind of personal qualities do you think he/she has that can help him/her cope with critical situations? / What kind of personal qualities do you think he/she lacks that make it difficult for him/her to cope with critical situations?
8. We hope to invite them to participate in our subsequent interviews. Would you be convenient to ask if they are willing to participate in our interviews?

**Part 2: Interviews with the Recommended**

Basic Interview Information (Can be filled in by the interviewer)

Interview Time:

Interview Location:

Interview Method (Online/Offline):

**(1) Interview Opening Remarks**

Hello, we are a psychological research team from [blinded for review]. Currently, we are conducting a survey on psychological resilience and stress - coping. Next, we will conduct an interview lasting about 40 minutes. The content of the interview will be kept strictly confidential. Thank you for sharing your experiences and feelings in stress - coping. We will record the interview, and all results will only be used for scientific research without involving your personal information. To ensure the effectiveness of the interview, please answer each question truthfully. If you have no questions, let's start!

**(2) Basic Information**

| Name |  | Gender |  |
| --- | --- | --- | --- |
| Ethnicity |  | Date of birth |  |
| Age |  | Education |  |
| What kind of occupation are you currently engaged in |  | | |
| What other occupations have you engaged in? |  | | |

**(3) Peer Evaluation: Insights into Elements of Resilience**

1. What kind of people do you think have strong stress - resistance ability when facing prolonged stress?

2. What are the characteristics of an "indomitable person" when facing prolonged stress? What kind of personal qualities do you think can help an individual cope with prolonged stress?

3. What external factors (such as family support, friend help, etc.) are conducive to coping with prolonged stress?

4. If you don't know a person and only have half an hour, what aspects of a person's performance will make you think he/she has strong stress - resistance ability in the face of prolonged stress? You can set up a practical scenario to assess the person or ask the person some questions. Feel free to share your ideas.

**(4) Self Evaluation**

1. As a **(occupation), please talk about the stress you have encountered in your work/study and life over the long term.

2. Do you think you have rich life experiences and have experienced many things?

3. How much stress do you usually have? How do you view stress? What impact has stress had on you?

4. When you are in a prolonged stress state, what are your performance and results?

5. Can you specifically mention which events caused the prolonged stress at that time? How did you cope with it at that time?

6. Will prolonged stress have a negative impact on you? What are the specific manifestations?

7. When you experience prolonged stress and for a period of time afterwards, what will you think and do? Please give at least one specific event.

**The Affect Regulation-based Resilience Scale (ARRS)**

**Guideline**

The following questions are aimed at understanding your attitude towards life, your mind, and reaction habits to stressful events. Please respond as quickly as possible to whether the statement is consistent with your actual situation and opinion. You do not have to think for a long time for each question, and there are no right or wrong answers.

5-point scale: Completely inconsistent; Relative inconsistent; Not sure; Relative consistent; Completely consistent

**Dimension 1 Inner Resources and Goal Orientation-9 questions**

1 After making a plan, I can execute it.

2 There are many people who have enlightened and helped me in life.

3 I spend most of my life doing things that make sense.

4 I am a person with faith inside.

5 I feel full within myself.

6 I know what I want.

7 What I am determined to do, I can usually find a way to do the same.

8 I have plenty of energy to cope when faced with big things.

9 I will work hard and persevere to achieve my goals.

**Dimension 2 Positive stress mindset-6 questions**

10 I like to do things that are challenging.

11 Stress can stimulate my potential.

12 Even in the face of adversity, I still have confidence in life.

13 After making a mistake, I can calm down and deal with the problem.

14 I can think in a focused manner when I encounter stressful events.

15 When I encounter a stressful event, I am able to look at the long term and do not care about short term gains or losses.

**Dimension 3 Self and Life Evaluation-9 questions**

16 I often complain about others.

17 I feel that bad things always happen to me.

18 I feel that life has been cheating me.

19 I spend my life in depression (e.g. suppressing emotions).

20 I hate myself.

21 I feel despair when I see my colleagues/classmates/neighbours etc. so good.

22 I don't think I have any merits to be proud of.

23 I feel that I can hardly escape from bad luck.

24 I feel that life owes me a lot.

**Dimension 4 Sensitivity-10 questions**

25 All kinds of nervous performance under pressure make me feel very humiliated.

26 I often do things I don't want to do, and I'm angry inside.

27 When I encounter something big, I feel overwhelmed, my mouth is dry and I have a cold sweat.

28 If a thing can't be solved quickly, I will quickly lose patience.

29 If I am already under a lot of pressure and then something urgent happens suddenly, I will be very broken.

30 I often worry about making mistakes.

31 Failing important things such as exams/interviews/competitions gets in the way of other things in my life.

32 I will keep blaming myself for saying or doing the wrong thing.

33 I feel overwhelmed when I encounter trouble.

34 When something bad happens, I don't want to face and admit it.
